# Cognitive deficit is correlated with sleep stability in insomnia: A cardiopulmonary coupling study

**DOI:** 10.1002/brb3.2068

**Published:** 2021-05-07

**Authors:** Xuan Zhang, Bingxin Song, Yanyan Liu, Yahui Wan, Kaili Zhou, Rong Xue

**Affiliations:** ^1^ Department of Neurology Tianjin Medical University General Hospital Airport Site Tianjin China; ^2^ Department of Neurology Tianjin Medical University General Hospital Tianjin China

**Keywords:** cardiopulmonary coupling analysis, insomnia, neuropsychological performance, sleep stability

## Abstract

**Objectives:**

To assess the correlation of cognitive function with sleep stability and depressive‐anxious symptoms in insomnia patients.

**Methods:**

Twenty‐two insomnia patients with cognitive impairment (insomnia‐CI), 21 insomnia patients with normal cognition (insomnia‐CN), and 15 matched healthy control subjects (HCs) were enrolled and completed neuropsychological tests, the Hamilton Depression and Anxiety Scales (HAMD and HAMA), the Epworth Sleepiness Scale, the Pittsburgh Sleep Quality Index (PSQI),the Insomnia Severity Index (ISI), and the cardiopulmonary coupling (CPC) examination. Ratios of high‐frequency coupling (HFC), low‐frequency coupling (LFC), and very low‐frequency coupling (VLFC) measured by CPC analysis represent stable sleep, unstable sleep, and wake/rapid eye movement (REM) sleep, respectively.

**Results:**

The HAMD, HAMA, PSQI, and ISI scores were higher in the insomnia‐CN patients than in the HCs (all *p* < .01). However, no differences were found in the HFC, LFC, and VLFC ratio between the HCs and insomnia‐CN groups. Compared with the insomnia‐CN patients, insomnia‐CI patients exhibited higher scores on the HAMD, HAMA (all *p* < .01), and PSQI (*p* < .05), performed worse on the Auditory Verbal Learning Test, Trial Making Test B, and Stroop Test B (all *p* < .01), had a lower HFC ratio, and had a higher LFC ratio in the CPC analysis (all *p* < .01). Furthermore, in the insomnia patients, poorer cognition was correlated with a decreased HFC ratio and an increased VLFC ratio (*r* = .356, *p* = .019; *r* = −.339, *p* =.026, respectively) and increased HAMD and HAMA scores (*r* = −.507, *p* < .001; *r* = −.561, *p* < .001, respectively); a higher VLFC ratio was correlated with an increased ISI score (*r* = .346, *p* = .023).

**Conclusions:**

Cognitive deterioration in insomnia patients was associated with a decreased stable sleep ratio, an increased wake/REM sleep ratio and more severe symptoms of depression and anxiety. CPC analysis can reflect the severity of insomnia.

## Graphical abstract

Sleep stability and mood are valuable therapy targets to potentially improve cognitive outcomes in the insomnia patients. The cardiopulmonary coupling (CPC) analysis is a practical measure for objective sleep evaluation, which is helpful to identify the insomnia patients with high risk of cognitive impairment.

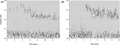

## 1 INTRODUCTION

Insomnia is characterized by subjective complaints of poor sleep quality or quantity despite adequate time for sleep, resulting in daytime fatigue, depressed mood, decreased cognition, lack of interest in social activity, and poor quality of life. An investigation in Swiss primary care showed that one‐third of patients reported insomnia symptoms, and 11% met the criteria for chronic insomnia in the Diagnostic and Statistical Manual of Mental Disorders, Fifth Edition (DSM‐5) (Maire et al., 2020). Insomnia increases the risk of various medical and psychiatric disorders, such as cardiovascular disease, stroke, cognitive impairment, depression, and anxiety disorders (Sivertsen et al., 2014).

Insomnia patients mostly complain of decreased memory or poor attention. Patients with chronic insomnia show poor performance on attention, intelligence, and memory tests (Lu et al., 2019). Clinically significant alterations in attention and episodic memory were also found in individuals with primary insomnia (Fortier‐Brochu & Morin, 2014). However, it has been reported that subjects with primary insomnia do not show deficits on neuropsychological tests (Orff et al., 2007). Insomnia with and without cognitive impairment (CI) may represent two different subtypes, between which the clinical and sleep differences remain unknown. Insomnia patients with short sleep durations present significant cognitive deficits (Fernandez‐Mendoza, 2017; Vgontzas et al., 2013). The anxiety‐depression triggered by the internalization of emotions predisposes the individual to insomnia, and this, in turn, intensifies depression. Anxiety‐depression may also exacerbate cognitive impairment in insomnia patients. Further research is needed to confirm the factors related to cognitive deficits in insomnia patients.

The cardiopulmonary coupling (CPC) method was proposed by the team of Thomas RJ (Thomas et al., 2005) and has been used as a new technique for stable and unstable sleep monitoring. In CPC method, electrocardiogram (ECG) is recorded, from which ECG‐derived respiratory (EDR) signal is deducted. The degree of coherent coupling between HRV and variations in the R‐wave amplitude caused by modulations of the respiratory tidal volume is quantified to generate frequency maps of coupled autonomic–respiratory oscillations. Although polysomnography (PSG) is the gold standard for the diagnosis of sleep disorders, it has limitations in availability, cost, and access and may not replicate normal sleep patterns in the home. The CPC technique is portable and can avoid the first night effect, which is usually seen in PSG examination. Thus, the CPC technique has been well applied in clinical and scientific research, especially in the objective sleep evaluation of insomnia patients. The high‐frequency coupling ratio in the CPC is related to the amount of slow‐wave oscillations in the EEG (Thomas et al., 2014). The cardiopulmonary coupling spectrogram is an ambulatory clinical biomarker of sleep stability and quality in health, sleep apnea, and insomnia (Thomas et al., 2018).

It has been reported that slow‐wave sleep has a beneficial effect on working memory (Ferrarelli., 2019), executive function (Diep et al., 2020), executive control of attention (Li et al., 2016), and processing speed (Della et al., 2018). Deficits in attention and episodic memory appear to be associated with sleep continuity and sleep microstructure in patients with insomnia (Fortier‐Brochu & Morin, 2014). The proportion of stable sleep is positively correlated with memory in depressed patients (Zhu et al., 2020). However, the question of whether stable or unstable sleep measured by CPC analysis correlates with cognitive impairment in insomnia patients remains unanswered. Therefore, the aim of this study was to (a) compare the clinical characteristics and sleep parameters evaluated by CPC among insomnia patients with and without CI and healthy controls (HCs); (b) determine the factors related to cognitive impairment in insomnia patients; and (c) evaluate the correlation of CPC variables with subjective complaints in insomnia patients.

## 2 PATIENTS AND METHODS

### 2.1 Subjects

Participants were recruited from Tianjin Medical University General Hospital from October 2017 to May 2019. The insomnia patients were diagnosed according to the standards described in the DSM‐5. Patients with the following conditions were excluded: (a) hearing loss, parachromatism, illiteracy, and the inability to complete the required tests; (b) a history of psychiatric diseases such as depressive disorder and schizophrenia; (c) severe medical conditions; (d) cerebrovascular disease, neurological conditions, a history of head trauma, or any other sleep disorder such as parasomnia or obstructive sleep apnea syndrome; and (e) treatment with a drug known to influence sleep. Seventy individuals with insomnia and 23 age‐, sex‐, and education‐matched healthy controls were enrolled. All of the participants signed agreement forms before the study. The study was approved by the Tianjin Medical University General Hospital Review Board and Ethics Committee.

### 2.2 CPC examination

Seventy individuals with insomnia and 23 HCs accepted the CPC examination at home. Participants were instructed to go to sleep at their usual bedtimes. The sleep data were collected by a novel ECG‐based home sleep monitoring device (AECG‐600D), which collected ECG, actigraphy, and body position data at a sampling rate of 128 Hz. Then, the data were stored in the Alibaba cloud system (Alibaba Cloud Computing Co. Ltd.). The CPC algorithm (The Sleep Quality Assessment System of CPC, V2.0) uses continuous ECG data and extracts heart rate variability and EDR activity from the ECG signal. The detailed original methodology of the CPC algorithm has been published (Thomas et al., 2005) and is available online at www.journalsleep.org. The CPC analyzer generated values for the following bandwidths of 0.1–0.4 Hz, 0.01–0.1 Hz, and 0.0039–0.01 Hz for high‐frequency coupling (HFC), low‐frequency coupling (LFC), and very low‐frequency coupling (VLFC), which represent stable sleep, unstable sleep, wake or rapid eye movement (REM) sleep, respectively. The apnea‐hypopnea index (AHI) was calculated by the time duration and average frequency of LFC. Twenty‐seven individuals with insomnia and 8 HCs whose AHI>5 were excluded. Forty‐three individuals with insomnia and 15 HCs were enrolled.

### 2.3 Subjective sleep and neuropsychological evaluations

Subjective sleep and neuropsychological evaluations were performed by a trained doctor in an evaluator‐blinded fashion. The sleep evaluation included the following: (a) daily sleepiness was evaluated with the Epworth Sleepiness Scale; (b) subjective sleep quality was evaluated with the Pittsburgh Sleep Quality Index (PSQI); (c) severity of insomnia was evaluated with the Insomnia Severity Index (ISI); and (d) severity of depressive and anxious symptoms was evaluated with the HAMD and HAMA. The neuropsychological evaluations included the following: (a) general cognitive functioning was evaluated with the Montreal Cognitive Assessment (MoCA) (Nasreddine et al., 2005); (b) verbal memory was evaluated with the Auditory Verbal Learning Test (AVLT), including the scores of total, short delay recall, long delay recall, and recognition (Guo et al., 2009); and (c) attention‐executive function was evaluated with the Trail Making Test (TMT) A, TMT B, Stroop Color‐Word Test (modified version) (Stroop A, Stroop B, and Stroop C) (Strauss et al., 2006). Patients with insomnia were divided into two groups according to the MoCA score (insomnia with normal cognition: MoCA score ≥ 26; insomnia with cognitive impairment: MoCA score < 26).

### 2.4 Statistical analysis

The results are expressed as the mean ± standard deviation (*SD*) for continuous variables and as the probability (percent) for categorical variables. Differences in the demographic, clinical, and sleep characteristics among the three groups were compared descriptively using chi‐square tests for categorical measures, using one‐way ANOVA tests for data that were normally distributed and which homoscedasticity was respected, using nonparametric Kruskal–Wallis or Mann–Whitney U tests for variables that were not distributed normally or for which homoscedasticity was not respected. Linear models were applied to compute the Pearson correlation coefficient (*r*) between scores of subjective sleep scales and CPC parameters, as well as cognition and CPC parameters and severity of depressive and anxious symptoms in the insomnia group. Statistical significance was defined as *p* < .05. The statistical analyses were performed using the SPSS software (Inc. Chicago, IL, USA). Figures were created using GraphPad Prism Version 5 (San Diego, USA).

## 3 RESULTS

### 3.1 Demographics, self‐report questionnaires, and neuropsychological evaluations of the participants

The demographic and clinical characteristics are reported in Table 1. No differences were found in age, gender, education, caffeine, or alcohol consumption among the three groups. Subjects in the insomnia‐CI and insomnia‐CN groups were significantly more likely to have a history of insomnia than those in the HC group (*p* < .01, *p* < .05, respectively).

**TABLE 1:** Demographic, clinical characteristics, and neuropsychological tests of the participants

	Insomnia‐CI (*n* = 22) (A)	Insomnia‐CN (*n* = 21) (B)	HCs (*n* = 15) (C)	*p*
Age, years	57.82 ± 7.37	54.14 ± 7.86	53.6 ± 8.85	NS
Female, number (%)	13 (59.1)	12 (57.1)	9 (60.0)	NS
Education, years	12.18 ± 3.28	13.95 ± 3.07	14.27 ± 3.20	NS
Caffeine drinker, number (%)	5 (22.7)	4 (19.0)	2 (13.3)	NS
Alcohol drinker, number (%)	4 (18.2)	5 (23.8)	2 (13.3)	NS
History of insomnia, number (%)	9 (40.9)	7 (33.3)	0 (0.0)	A > C^a^, B > C^b^
HAMD	12.73 ± 5.79	8.33 ± 4.12	3.00 ± 2.17	A > B^a^, B > C^a^
HAMA	12.68 ± 6.14	8.33 ± 4.46	2.40 ± 1.99	A > B^a^, B > C^a^
MoCA	19.95 ± 3.42	27.24 ± 1.22	26.40 ± 2.85	A < B^a^, A < C^a^
**Verbal memory function**				
AVLT				
Total	14.68 ± 3.91	18.52 ± 4.19	20.40 ± 4.58	A < B^a^, A < C^a^
Short delay memory	4.50 ± 2.63	6.67 ± 2.27	7.00 ± 2.17	A < B^a^, A < C^a^
Long delay memory	4.05 ± 2.79	6.81 ± 2.36	7.53 ± 2.42	A < B^a^, A < C^a^
Recognition	10.18 ± 1.89	10.90 ± 1.67	11.73 ± 3.88	NS
**Attention‐executive function**				
TMT				
TMT A	60.75 ± 26.36	49.33 ± 12.09	48.98 ± 24.31	NS
TMT B	201.53 ± 80.29	148.75 ± 41.08	124.10 ± 40.06	A > B^a^, A > C^a^
Stroop test				
Stroop A	17.26 ± 7.16	14.42 ± 3.18	14.08 ± 1.97	NS
Stroop B	23.31 ± 5.97	19.24 ± 3.86	18.44 ± 2.89	A > B^a^, A > C^a^
Stroop C	32.80 ± 9.25	30.79 ± 7.00	30.96 ± 8.94	NS

Note

Plus‐minus values are means ± *SD*.

Abbreviations: AVLT, Auditory Verbal Learning Test; HAMA, Hamilton Anxiety Scale; HAMD, Hamilton Depression Scale; HCs, healthy controls; Insomnia‐CI, Insomnia with cognitive impairment; Insomnia‐CN, Insomnia with normal cognition; MoCA, Montreal Cognitive Assessment; TMT, Trial Making Test.

^a^ *p* < .01;

^b^ *p* < .05.

Compared with the HCs, the insomnia‐CN patients exhibited more severe symptoms of depression and anxiety (all *p* < .01). Compared with the insomnia‐CN group, the insomnia‐CI patients exhibited more severe symptoms of depression and anxiety (all *p* < .01). Compared with the insomnia‐CN patients, the insomnia‐CI patients performed worse in verbal memory: AVLT total (*p* < .01), AVLT short delay recall (*p* < .01), AVLT long delay recall (*p* < .01); and attention‐executive function: TMT B (*p* < .01), Stroop Test B (*p* < .01).

### 3.2 Objective and subjective sleep characteristics of insomnia patients with and without CI

**FIGURE 1:**
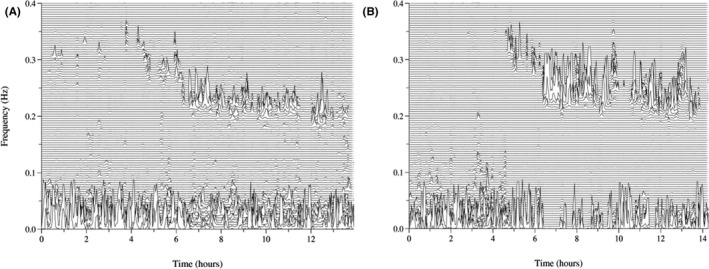
Sleep spectrums of insomnia and healthy subjects. (a) sleep spectrum of a patient with insomnia; (b) sleep spectrum of a healthy subject

Sleep spectrums of insomnia and healthy subjects are shown in Figure 1. No differences were found in the objective sleep parameters in CPC analysis, such as sleep efficiency, HFC (stable sleep) ratio, LFC (unstable sleep) ratio, VLFC (wake/REM sleep) ratio and AHI, between the insomnia‐CN patients and HCs. The sleep latency in the insomnia‐CI patients and insomnia‐CN patients was longer than that in the HCs. The HFC (stable sleep) ratio in the insomnia‐CI patients was significantly lower than that in the insomnia‐CN patients (*p* < .01). The LFC (unstable sleep) ratio in the insomnia‐CI patients was significantly higher than that in the insomnia‐CN patients (*p* < .01). The VLFC (wake/REM sleep) ratio in the insomnia‐CI patients was significantly higher than that in the HCs (*p* < .01). The sleep efficiency in the insomnia‐CI patients was significantly lower than that in the HCs (*p* < .05). There was no significant difference in the total sleep time or AHI among the three groups.

The insomnia‐CI and insomnia‐CN patients suffered from poorer sleep quality (all *p* < .01) and more severe insomnia (all *p* < .01) in the subjective sleep evaluations than the HCs. The insomnia‐CI patients suffered from poorer subjective sleep quality than the insomnia‐CN patients (*p* < .05). The details of sleep characteristics are summarized in Table 2.

**TABLE 2:** Objective and subjective sleep characteristics of the participants

	Insomnia‐CI (*n* = 22)(A)	Insomnia‐CN (*n* = 21)(B)	HCs (*n* = 15)(C)	*p*
**Objective sleep evaluation (CPC)**				
Total sleep time, hours	6.75 ± 1.62	6.98 ± 1.07	7.54 ± 0.67	NS
Sleep latency, minutes	34.68 ± 21	29.87 ± 20	9.33 ± 7.02	A > C^a^, B > C^a^
Sleep efficiency, %	72.66 ± 7.49	79.30 ± 10.11	83.39 ± 4.53	A < C^b^
HFC (stable sleep), %	43.23 ± 14.61	54.16 ± 14.01	61.90 ± 9.63	A < B^a^, A < C^a^
LFC (unstable sleep), %	33.27 ± 10.63	25.17 ± 8.74	22.34 ± 7.45	A > B^a^, A > C^a^
VLFC (wake/REM sleep), %	24.13 ± 7.13	20.87 ± 7.53	16.30 ± 6.72	A > C^a^
AHI	1.17 ± 1.38	1.09 ± 1.45	0.89 ± 1.07	NS
**Subjective sleep scales**				
ESS	2.95 ± 2.16	3.07 ± 2.37	2.86 ± 2.25	NS
PSQI	15.41 ± 3.02	13.24 ± 3.21	3.93 ± 2.25	A > B^b^, B > C^a^
ISI	15.55 ± 5.43	12.76 ± 6.10	3.60 ± 3.18	A > C^a^, B > C^a^

Note

Plus‐minus values are means ± *SD*.

Abbreviations: AHI, apnea‐hypopnea index; CPC, cardiopulmonary coupling; ESS, Epworth Sleepiness Scale; HCs, healthy controls; HFC, high‐frequency coupling; Insomnia‐CI, Insomnia with cognitive impairment; Insomnia‐CN, Insomnia with normal cognition; ISI, Insomnia Severity Index; LFC, low‐frequency coupling; NS, not significant; PSQI, Pittsburgh Sleep Quality Index; REM, rapid eye movement; VLFC, very low‐frequency coupling.

^a^ *p* < .01;

^b^ *p* < .05.

### 3.3 Correlation between cognition and CPC variables, severity of depression and anxiety in the insomnia group

**FIGURE 2:**
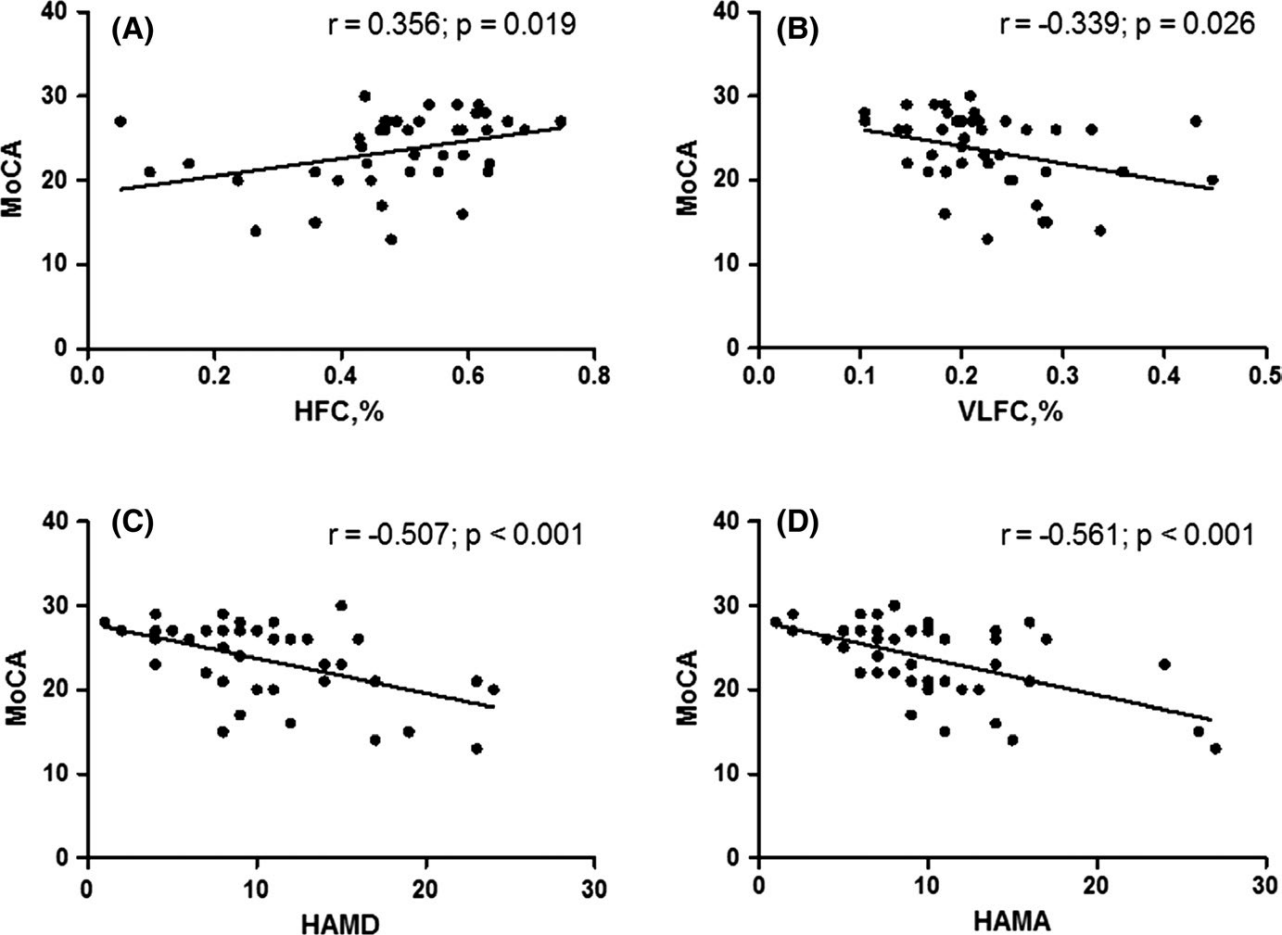
Correlation between cognition and CPC variables, severity of depressive, and anxious symptoms in the insomnia group. In the insomnia group, the MoCA score showed a positive correlation with the HFC (stable sleep) ratio (*r* = .356, *p* =.019) (a), a negative correlation with the VLFC (wake/REM sleep) ratio (*r* = −.339, *p* =.026) (b), a negative correlation with the HAMD score (*r* = −.507, *p* < .001)(c), and a negative correlation with the HAMA score (*r* = −.561, *p* < .001) (d). HAMA = Hamilton Anxiety Scale; HAMD = Hamilton Depression Scale; HFC = high‐frequency coupling; LFC = low‐frequency coupling; MoCA = Montreal Cognitive Assessment; REM = rapid eye movement; VLFC = very low‐frequency coupling

In the insomnia group, the MoCA score showed a positive correlation with the HFC (stable sleep) ratio (*r* = .356, *p* = .019), a negative correlation with the VLFC (wake/REM sleep) ratio (*r* = −.339, *p* =.026), a negative correlation with the HAMD score (*r* = −0.507, *p* < .001), and a negative correlation with the HAMA score (*r* = −.561, *p* < .001) (Figure 2).

### 3.4 Correlation between scores of subjective sleep evaluation and CPC variables in the insomnia group

**FIGURE 3:**
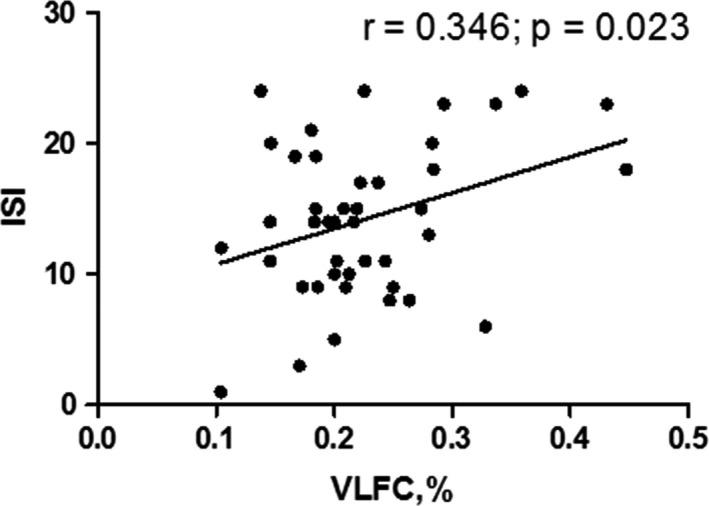
Correlation between scores of subjective sleep evaluation and CPC variables in the insomnia group. The ISI score was correlated with the VLFC (wake/REM sleep) ratio positively in the insomnia group (*r* = .346, *p* = .023). REM, rapid eye movement; VLFC, very low‐frequency coupling

The ISI score was positively correlated with the VLFC (wake/REM sleep) ratio in the insomnia group (*r* = .346, *p* = .023) (Figure 3). No correlation was found between the ESS, PSQI, and CPC variables in the insomnia group.

## 4 DISCUSSION

The key findings of our study are as follows: (a) compared with insomnia‐CN patients, insomnia‐CI patients showed a lower HFC (stable sleep) ratio, a higher LFC (unstable sleep) ratio, and more severe symptoms of depression and anxiety; (b) poorer cognition in insomnia patients was associated with a decreased HFC (stable sleep) ratio, an increased VLFC (wake/REM sleep) ratio measured by CPC analysis and more severe symptoms of depression and anxiety; and (c) the insomnia severity index was positively correlated with the VLFC (wake/REM sleep) ratio in the insomnia group. CPC analysis can reflect the severity of insomnia and may be helpful for identifying severe insomnia patients with cognitive dysfunction.

In our study, the cognitive function of some individuals with insomnia was preserved, while some individuals with insomnia suffered from cognitive decline, especially in verbal memory and attention‐executive function. Although insomnia patients always complain of memory decline or poor attention in daily life, previous studies on cognitive deficits in insomnia patients are controversial. Most studies have supported that insomnia patients perform worse on neuropsychological tests than healthy controls. Insomniacs with cognitive decline exhibit performance impairments for several cognitive functions, including working memory, episodic memory, complex attention, alertness, and executive function (de Almondes et al., 2020; Ballesio et al., 2019; Fortier‐Brochu et al., 2012; Wardle‐Pinkston et al., 2019). The insomnia‐CI patients in our study exhibited similar domains of cognitive deficits as those in previous studies. However, some primary insomnia patients did not show deficits on neuropsychological tests (Orff et al., 2007). Insomnia patients who were nontreatment seekers exhibited subjective cognitive impairment but no objective impairment on testing, while treatment seekers exhibited significant objective impairment (Goldman‐Mellor et al., 2015). It was observed that insomnia patients with normal objective sleep duration performed better than those with short sleep duration (Fan et al., 2019). The discrepant results may be ascribed to diverse factors, such as the diverse clinical phenotypes of insomnia (Kyle et al., 2017), various mood states, sleep continuity, sleep microstructure, dysfunctional beliefs (Fortier‐Brochu & Morin, 2014), objective sleep duration (Fan et al., 2019), slow‐wave sleep (Li et al., 2016). Therefore, it is meaningful to identify insomnia patients with cognitive impairment and the factors that contribute to cognitive deterioration for further targeted therapy.

In our study, the insomnia‐CI patients showed a significantly decreased HFC (stable sleep) ratio and increased LFC (unstable sleep) ratio measured by CPC analysis compared with the insomnia‐CN patients. Moreover, poorer cognition in insomnia patients was associated with a decreased HFC (stable sleep) ratio and an increased VLFC (wake/REM sleep) ratio. The results indicated that stable sleep played a pivotal role in preserving cognition, which was also supported by previous studies. The proportion of stable sleep is positively correlated with immediate and delayed memory in depressed patients (Zhu et al., 2020). Delta power, which is characteristic of slow‐wave sleep (SWS), was correlated with stable sleep in the CPC analysis (Thomas et al., 2014). Thus, as a practical and portable device that objectively measures the proportions of stable and unstable sleep, the CPC technique may be helpful for identifying severe insomnia patients with cognitive dysfunction. SWS is the stage during which synaptic activity is minimal and clearance of neuronal metabolites is high (Varga et al., 2016). An increasing number of studies have proven that sleep loss, especially SWS loss, increases the risk of cognitive impairment. Reduced and fragmented SWS was associated with increases in CSF Aβ‐42 (Ju et al., 2017; Varga et al., 2016) and accumulation of tau aggregates. Reports of shorter sleep duration and poorer sleep quality were associated with greater A‐β burden evaluated by PET (Spira et al., 2013). Chronic sleep deprivation accelerated the spread of tau protein aggregates in neural networks (Wang & Holtzman, 2020). In addition, impaired synaptic plasticity, increased inflammation, and neurotransmitter changes are potential mechanisms linking sleep disturbances and cognition (Yaffe et al., 2014).

Our study showed that insomnia‐CI patients suffered from more severe symptoms of depression and anxiety than insomnia‐CN patients, and cognitive decline was correlated with the severity of depressive and anxious symptoms in insomnia patients. It is known that cognitive impairment is a symptomatic domain identified across many mental disorders. However, few studies have focused on the role of depressive‐anxious symptoms on cognition between insomnia‐CI and insomnia‐CN patients, who did not meet the criteria of depression disorder or generalized anxiety disorder. It was reported that 36.8% of chronic insomnia patients combined with depression (Maire et al., 2020). Consistent with our study, depressive symptomatology was associated with cognitive deterioration in institutionalized older adults (Camacho‐Conde & Galan‐Lopez, 2020). In older adults with insomnia and a history of chronic pain, improving sleep may benefit lower level cognition, whereas reducing depression may affect higher level cognition (Curtis et al., 2018). Cortical amyloid moderated the association between worsening depressive symptoms and declining cognition in older adults (Gatchel et al., 2019). The likely biological mechanisms linking depression to dementia include vascular disease, alterations in glucocorticoid steroid levels and hippocampal atrophy, increased deposition of amyloid‐β plaques, inflammatory changes, and deficits of nerve growth factors (Byers & Yaffe, 2011). There are bidirectional relationships between sleep and depressive‐anxious symptoms, which contribute to the cognitive deterioration of insomnia patients.

The ISI score was positively correlated with the VLFC (wake/REM sleep) ratio in the insomnia group, which indicated that the VLFC ratio of the CPC technique is an objective indicator to reflect the severity of insomnia. Insomnia patients with more wake/REM sleep may perceive that they do not sleep well; thus, they are unsatisfied with their sleep and complain about the symptoms of insomnia bitterly. It was demonstrated that CPC analysis was fit for differentiating and objectively quantifying the sleep quality of insomnia patients (Schramm et al., 2013). The PSQI score showed a negative correlation with the HFC (stable sleep) ratio in PD patients (Chen et al., 2018); however, no correlation was found between the PSQI score and sleep parameters of CPC analysis in insomnia patients. Considering the existence of subjective insomnia, it is necessary to evaluate the objective sleep parameters of insomnia patients using the CPC technique, which is available and portable.

There were some limitations to our study. First, the sample size of the study was small. It is necessary to verify the findings in a larger sample. Second, PSG was not performed to determine the accurate proportions of sleep stage and their association with sleep parameters of CPC analysis. Third, it was difficult to differentiate the wake and REM sleep by CPC analysis. Further studies with a large sample and PSG examination should be conducted to obtain more reliable findings in the future.

## 5 CONCLUSION

Our study identified poorer performance in verbal memory and attention‐executive tests in insomnia‐CI patients. The insomnia‐CI patients showed a lower HFC (stable sleep) ratio and a higher LFC (unstable sleep) ratio measured by CPC analysis and reported more severe symptoms of depression and anxiety than the insomnia‐CN patients. Poorer cognition in insomnia patients was associated with a decreased HFC (stable sleep) ratio, an increased VLFC (wake/REM sleep) ratio, and more severe symptoms of depression and anxiety. Sleep stability and mood are valuable therapeutic targets to potentially improve cognitive outcomes in insomnia patients. The VLFC ratio of the CPC technique is an objective indicator to reflect the severity of insomnia. CPC analysis is a practical measure for objective sleep evaluation, which is helpful for identifying severe insomnia.

## CONFLICTS OF INTEREST

None.

## AUTHOR CONTRIBUTIONS

Guarantor of integrity of the entire study: Xuan Zhang and Rong Xue. Study concept: Xuan Zhang and Rong Xue. Study design: Xuan Zhang, Bingxin Song, Yanyan Liu and Rong Xue. Literature research: Yahui Wan and Kaili Zhou. Clinical assessment: Bingxin Song and Yanyan Liu. CPC examination: Bingxin Song and Yanyan Liu. Data analysis: Xuan Zhang and Bingxin Song. Manuscript preparation: Xuan Zhang and Bingxin Song. Manuscript review: Rong Xue.

### PEER REVIEW

The peer review history for this article is available at https://publons.com/publon/10.1002/brb3.2068.

## Data Availability

The data that support the findings of this study are available from the corresponding author upon reasonable request.
